# Experimental Study on Productivity Performance of Household Combined Thermal Power and Biogas System in Northwest China

**DOI:** 10.1155/2018/7420656

**Published:** 2018-05-13

**Authors:** Jian Kang, Jinping Li, Xiaofei Zhen, Yassir Idris Abdalla Osman, Rong Feng, Zetian Si

**Affiliations:** ^1^Western China Energy & Environment Research Center, Lanzhou University of Technology, Lanzhou 730050, China; ^2^School of Material Science and Engineering, Lanzhou University of Technology, Lanzhou 730050, China; ^3^Key Laboratory of Complementary Energy System of Biomass and Solar Energy, Lanzhou, Gansu Province 730050, China; ^4^Collaborative Innovation Center of Key Technology for Northwest Low Carbon Urbanization, Lanzhou 730050, China; ^5^Shaanxi Key Laboratory of Industrial Automation, Shaanxi University of Technology, Hanzhong 723000, China

## Abstract

Ample quantities of solar and local biomass energy are available in the rural regions of northwest China to satisfy the energy needs of farmers. In this work, low-temperature solar thermal collectors, photovoltaic solar power generators, and solar-powered thermostatic biogas digesters were combined to create a heat, electricity, and biogas cogeneration system and were experimentally studied through two buildings in a farming village in northwestern China. The results indicated that the floor heater had the best heating effect. And the fraction of the energy produced by the solar elements of the system was 60.3%. The photovoltaic power-generation system achieved photovoltaic (PV) conversion efficiencies of 8.3% and 8.1% during the first and second season, respectively. The intrinsic power consumption of the system was 143.4 kW·h, and 115.7 kW·h of electrical power was generated by the system in each season. The average volume of biogas produced daily was approximately 1.0 m^3^. Even though the ambient temperature reached −25°C, the temperature of the biogas digester was maintained at 27°C ± 2 for thermostatic fermentation. After optimization, the energy-saving rate improved from 66.2% to 85.5%. The installation reduced CO_2_ emissions by approximately 27.03 t, and the static payback period was 3.1 yr. Therefore, the system is highly economical, energy efficient, and beneficial for the environment.

## 1. Introduction

Nonrenewable energy production and utilization prevail in rural regions of northwest China due to restrictions imposed by geographical location, costs associated with clean energy implementation, and insufficient knowledge of deployment and operation of clean energy systems. The majority of these rural communities still rely on coal energy, and households have been found to be significant energy consumers [1]. In fact, CO_2_ emissions in rural residential areas have grown at higher per-capita rates than those of urban areas [2]. Notwithstanding these challenges, agricultural communities in northwest China are rich in renewable resources, especially solar energy and organic biomass, and sustainable generation of clean energy for rural communities has been found to be feasible [3]. In fact, harnessing these sources has been deemed necessary to achieve China's goals of carbon emission reduction and transition to clean energy, as well as eliminating rural energy poverty [4].

Several studies have investigated the performance of solar and biomass systems for energy production [4–11]. Bhattarai et al. [5] found that tank capacity has a significant effect on thermal efficiency and economic performance of photovoltaic and thermal solar (PV/T) collectors. Esen and Yuksel [6] used solar, geothermal energy, and biogas to heat a model greenhouse (6 m × 4 m × 2.10 m) in Turkey, successfully attaining a plant-friendly temperature of 23°C. Jenssen et al. [7] analyzed a model municipality in Germany that uses biomass energy to understand the balance between the reduction in CO_2_ emissions and the increase in land-use competition and energy supply costs. The findings of Jenssen et al. indicate that heat and power demand can be easily met with biomass, but transport fuel necessitates a different source. Aguilar et al. [12] assessed and implemented a pilot-scale, closed-loop system that combined a solar thermal collector, an anaerobic digester, and a constructed wetland treatment system that used organic wastes for energy production, in which the wastes were subsequently treated prior to their release into the environment. The implemented system showed that organic wastes can be efficiently used to produce energy, while protecting the environment. O. Ozgener and L. Ozgener [9] utilized a driveway as a solar thermal panel to enhance the efficiency of a solar-assisted geothermal heat pump system. The results are reported as a 68% energy replacement for the product/fuel of the entire system and for the driveway that is used as the solar collector. Chen et al. [10] performed experiments and numerical simulations to study a combined solar system consisting of a solar collector and a CO_2_ heat pump. The simulated results indicated that the optimized system could reduce electricity expenditure by 14.2% and improve solar energy production by 8%. The solar portion of the optimized system was 71.1%. Wu et al. [11] proposed an optimal energy management system for a grid-connected PV-battery hybrid system for optimal harnessing of solar energy to meet consumer demand.

The scale of energy supply systems based on renewable energy at home and abroad and that can meet the needs of multiple levels of energy use is often too large to be suitable for the highly dispersed characteristics of residential properties still existing in rural China. Photovoltaic power generation, low-temperature heat collection, and biomass anaerobic fermentation and production of biogas, as three mature technologies for renewable energy use, can meet the energy needs of rural households in northwest China, such as household electricity, thermal energy, and domestic gas. However, the single-technology renewable energy utilization devices have great limitations in terms of energy supply stability and meet the needs of multiple levels of energy use and are severely constrained by environmental factors; therefore, they can be integrated with current solar photovoltaic power-generation technologies and solar energy cryogenic sets. Thermal technology and solar-powered constant-temperature biogas digester technology are used to construct a household heat and electricity cogeneration system that uses solar energy and biomass energy as input and can meet the needs of farmers in multiple levels in northwest cold regions. The literature shows that the potential for a 100%-renewable energy supply using solar energy, biomass energy, or a combination thereof has been either predicted or observed in different settings. However, verification by complete deployment of said technologies in real scenarios and analyses of measurements of relevant energy parameters to assess efficiency and cost remain scarce. Here, we report findings from an experimental study that was conducted under actual operating conditions over two winter seasons. We studied a heating-electricity-gas cogeneration system for use in two inhabited buildings located in Zhangma village (Gansu Province, Minqin County, China), each covering an area of 117 m^2^. Radiator heaters were used during the first season and low-temperature floor heaters during the second season. Comparative analysis was performed of the power-generation performance of this system using different heating terminals. In addition, a comprehensive evaluation of the resulting energy savings and emission reductions was performed, together with an assessment of economic viability.

## 2. Materials and Methods

### 2.1. Experimental Energy-Generation System

The experimental heating-electricity-gas cogeneration system was installed in a single-block building in Zhangma village. The system comprised three subsystems: a combined heating subsystem using both solar energy and a coal-fired boiler, a PV solar-power-generation subsystem, and a solar-heated thermostatic biogas digester. The heating system comprised a coal-fired boiler, heat-dissipating terminals, a water-circulating pump, valves, pipes, and six sets of evacuated-tube solar collectors. The heat-dissipating terminals consisted of radiator heaters that were subsequently replaced by low-temperature floor heaters. Each set of solar collectors comprised 40 evacuated tubes of length 1.8 m and diameter 0.058 and a collector with a surface area of 3.85 m^2^. The solar collectors were connected in series. The power-generation subsystem consisted of an array of solar cells, a controlled inverter, and battery cells. The PV array consisted of 10 single-crystal silicon PV cells divided into five parallel sets (each set having two solar cells connected in parallel).

**Figure 1:**
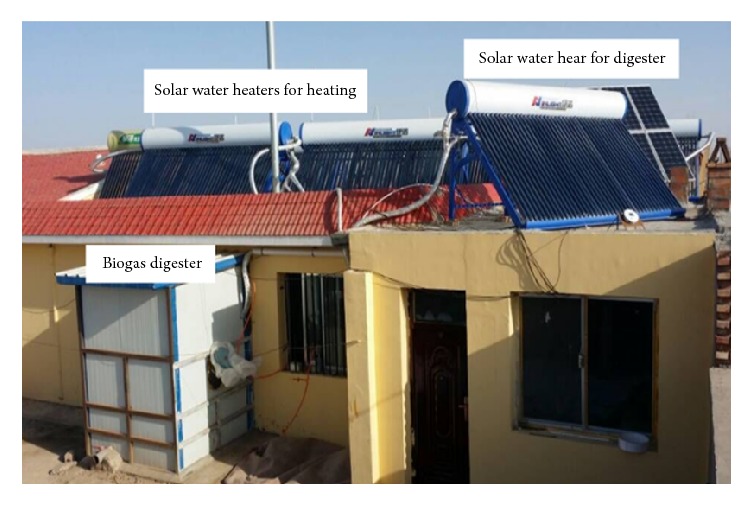
Photograph of the heat-electricity-biogas cogeneration system.

**Figure 2:**
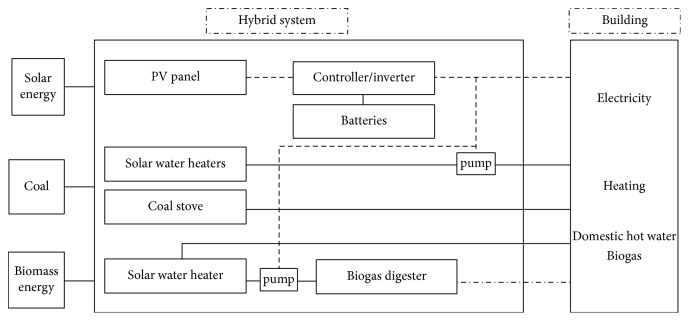
Integration schematic of the hybrid system.

The system included four batteries divided into two parallel sets, each set having two batteries connected in series. The total output power of this system was 1000 kW. The solar-heated thermostatic biogas digester subsystem was composed of a single set of evacuated-tube solar collectors, a biogas digester, heating coil, water-circulating pump, valves, and a red mud soft-matter biogas bag. Some of the power generated by the PV array was used to power the circulation pumps that drive hot water from the water tank into the building and into the biogas digester to provide heat. The remaining power supplied electricity for household usage. A photograph and schematic of the heat-electricity-gas cogeneration system are shown in Figures 1 and 2, respectively.

### 2.2. Experimental Parameters and Measuring Instruments

The two experimental periods were from December 1, 2014, to March 31, 2015, and from December 1, 2015, to March 31, 2016. The measurements and measuring instruments are shown in Table 1. All the parameters were automatically acquired and recorded using an Agilent 34970A data-acquisition device at a scanning interval of 10 s. An extraction pump was used at a fixed time each day to transfer the biogas produced within the fermentation bag into the gas storage bag. Daily gas production was measured by a G16 gas meter, and its composition was analyzed using a Gas 600 portable biogas analyzer. A DDS1531 single-phase electronic electricity meter was used to measure daily electricity consumption, which was then scaled to obtain daily coal consumption.

## 3. Methods

(1) Power generated by the PV array is expressed as

(1) $$\begin{array}{c} E=\sum UIt. \end{array}$$

This equation represents the power generated by the PV array, *E* (expressed in J). *U* is the PV array's output voltage (V), *I* is the array's output current (A), and *t* is the time (s).

(2) The quantity of heat provided to the building by the solar collectors is expressed as

(2) $$\begin{array}{c} Q=\sum cm\left(\;t_{in}-t_{out}\;\right)t. \end{array}$$

In (2), *Q* is the heat provided to the building by the solar collectors (J), *c* is the heat capacity of water (4200 J/kg·°C), *m* is the flow of the circulated water (kg/s), *t*_in_ is the supply water temperature (°C), *t*_out_ is the return water temperature (°C), and *t* is the time (s).

(3) The energy-saving rate (*η*) due to the energy-conserving measures taken by a user [13] is expressed as

(3) $$\begin{array}{c} \eta =\frac{W_{1}-W_{2}}{W_{1}}\times 100\%, \end{array}$$

where *W*_1_ and *W*_2_ represent energy consumption before and after adopting energy-saving measures, respectively. For this system, the energy-saving rate occurs by reducing consumption of standard coal.

## 4. Results and Discussion

### 4.1. Performance Analysis: Heating Stability of Solar-Powered Heating Subsystem

Winters in the northwestern regions of China are cold and dry, with significant diurnal temperature differences. Periods of extreme rain/snow are common. Therefore, auxiliary coal-fired boilers are required as a heating source in addition to a solar heater, to ensure the continuity and stability of the power-generation system. The heating system has three different heating modes that are used according to the availability of solar radiation: (1) with adequate sunlight, all heat is obtained from solar energy; (2) with weak sunlight, heating is provided by solar energy and the coal-fired boiler in combination; and (3) during rain, snow, and extreme weather events, all heat is provided by the coal-fired boiler. During the first winter (2014-2015), there were 82 d of clear weather, 26 d of cloudy weather, and 13 d of extreme rainy/snowy weather, compared with 70, 34, and 18 d, respectively, during the second winter (2015-2016). The numbers of days for each heating mode during both seasons are shown in Table 2. This comparison shows that the weather over the course of the second cool season was generally poorer, but that solar-powered heating alone was used on significantly more days than those requiring boiler usage. This indicates that, when the heating system was switched from radiators to low-temperature floor heating, the system showed greater resistance to weather-induced interference. In addition, the power-generation stability of the system improved significantly during these periods.

### 4.2. Indoor Temperatures

**Figure 3:**
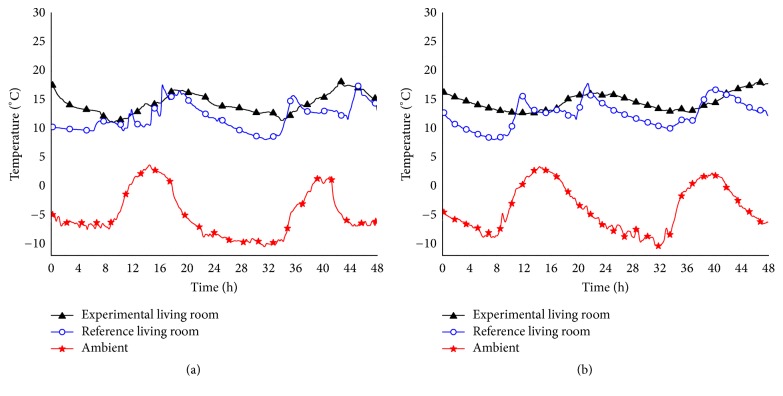
Ambient and indoor temperatures: (a) December 30 and 31, 2014; (b) December 2 and 3, 2015.

To compare the effectiveness of the three heating modes, 4 d with similar ambient temperatures were selected from each of the heating seasons, and the temperature data acquired from the building's living room were evaluated. There was ample solar radiation on those days, so solar energy was used to heat the experimental building, while a coal-fired boiler heated the reference building. Heating was provided between 16:00 h and 24:00 h each day. Indoor and ambient temperatures for the experimental and reference buildings on December 30 and 31, 2014, are shown in Figure 3(a). Ambient temperature ranged between −10.4°C and 3.8°C. The average living-room temperature was 14°C (range 7.3°C, minimum 11°C) in the experimental building, compared with 12°C (range 10.4°C, minimum 8°C) in the reference building.  Figure 3(b) illustrates the indoor and ambient temperatures of the experimental and reference buildings on December 2 and 3, 2015, during which time the ambient temperature ranged between −10.8°C and 3.2°C. The average living-room temperature was 14.3°C (minimum 12.4°C, range 4.4°C) in the experimental building, compared with 12.4°C (minimum 8.1°C, range 9.8°C) in the reference building. The living-room temperature of 14.3°C in the experimental building, which had been modified to conserve energy, met the requirements of the Design Standard for Energy Efficiency of Rural Residential Buildings (GBT50824-2013). In addition, the use of low-temperature floor heating resulted in the highest average indoor temperature and a smooth temperature-variation curve with minimal fluctuation. This mode of heating provided the greatest stability among the observed cases.

### 4.3. Indoor Relative Humidity

**Figure 4:**
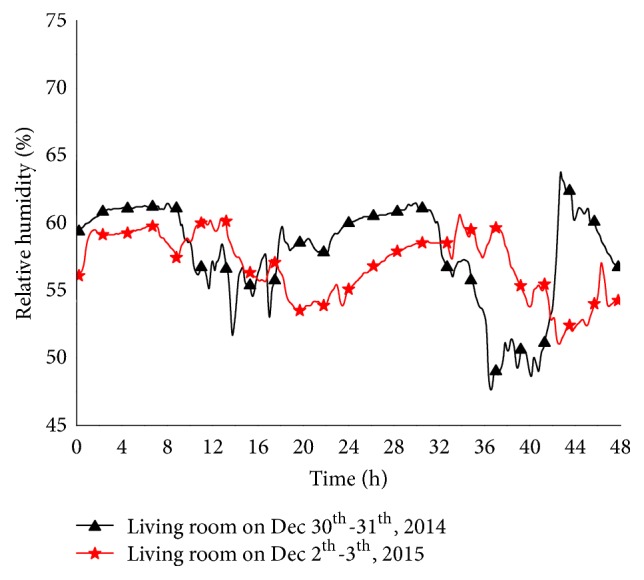
Relative humidity in the living room (experimental building) during December 30 and 31, 2014, and December 2 and 3, 2015.

Figure 4 shows relative humidity in the living room of the experimental building on December 30 and 31, 2014, and December 2 and 3, 2015. When the building was heated with solar-driven radiators, the relative humidity was 47–65%, compared with 51–60% when using solar-driven low-temperature floor heating. Both heaters were able to provide a comfortable range of relative humidity during the winter, ranging between 40% and 60% [14, 15]. However, the low-temperature floor heater resulted in more consistent humidity levels and was more stable and provided greater comfort than the solar-driven radiator.

### 4.4. Efficiency of Solar Collector

The efficiency of solar collectors is determined mainly by the quantity of solar radiation, temperature of the water tank, and ambient temperature. The average daily difference between the water tank temperature and ambient temperature and the cumulative solar radiation were treated as independent variables; the heat collected over the course of 1 d was treated as the dependent variable. The following equation was obtained using multiple linear regressions to describe the quantity of heat collected by a single solar collector over the course of 1 d:

(4) $$\begin{array}{c} Q_{s}=2.32E-0.30\left(\;\bar{T_{s}}-\bar{T_{e}}\;\right)+5.75, \end{array}$$

where *Q*_*s*_ is the quantity of heat collected by a single solar collector over the course of 1 d (expressed in MJ), *E* is the cumulative solar radiation (MJ/m^2^), *T*_*s*_ is the average daily temperature of the water tank (°C), and *T*_*e*_ is the average daily ambient temperature (°C).

The results of the data analysis are shown in Table 3. The multiple determination coefficient (*R*^2^ = 0.633) indicates a moderate fit, which is reasonable for the expected level of uncertainty in the observed process. The standard error was 0.332, representing an average error of 0.322 MJ in predicting the average daily difference between the water tank temperature and ambient temperature, the heat collected by the solar collector over the course of 1 d, and cumulative solar radiation. This error might be due to environmental factors that were not accounted for in this study, such as dust and wind speed. The regression coefficient *β*_1_ was 2.32, indicating that the quantity of heat collected by a collector over the course of 1 d will increase by 2.32 MJ for every 1-MJ increase in cumulative solar radiation, assuming that the difference between average water tank temperature and ambient temperature remains constant. The regression coefficient *β*2 was −0.30, which indicates that the heat collected by a collector over the course of 1 d decreases by 0.30 MJ for each 1°C increase in the average difference between water tank temperature and ambient temperature, assuming that cumulative solar radiation remains unchanged.

**Figure 5:**
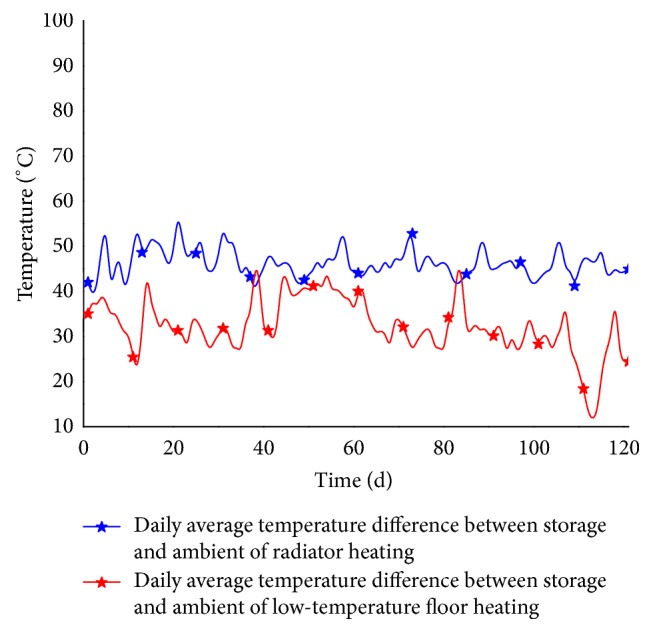
Daily average temperature difference between water tank temperature and ambient temperature during two heating seasons.

By dividing both sides of (4) by *A*_1_*E* (where *A*_1_ is the area of a single solar collector, 3.85 m^2^), we then obtain the equation for calculating the collector's daily average collection efficiency,

(5) $$\begin{array}{c} \bar{\eta }=0.60-\frac{0.078\left(\bar{T_{s}}-\bar{T_{e}}\right)-1.494}{E}, \end{array}$$

from which it can be shown that decreases in the average difference between the water tank temperature and ambient temperature will increase the collector's daily average collection efficiency, if cumulative solar radiation remains unchanged.  Figure 5 shows that this difference was generally smaller when using the floor heater compared with the radiator heater. Therefore, the use of floor heaters is beneficial for increasing the average daily collection efficiency.

## 5. Analysis of Solar Fraction of Solar-Powered Heating System

The solar fraction *f* refers to the ratio of heat provided by the solar energy system versus the required heating load. Using (2), the total heat supplied by solar energy during the two heating seasons was calculated as 11619 MJ and 22715 MJ, respectively. The required heating load of the system was calculated as follows.

(1) The experimental building had a total footprint of 117 m^2^; the radiator and floor heaters were located in the three bedrooms and living room, giving an effective heating area of 64 m^2^. The heat consumption of the building envelope, *Q*_HT_, calculated using (6), was 3328.6 W. The details of this calculation are shown in Table 4.

(2) The heat loss by infiltration can be calculated as follows:

(6) QINF=0.28×1.293×0.5×173×14+2.6=519.9 W.

(3) The indoor heating of the building is given by

(7) $$\begin{array}{c} Q_{IH}=64.4\times 3.8=244.7\;W. \end{array}$$

Based on (5), the heat consumption of the building, *Q*_*H*_, was 3603.8 W. Therefore, the required heating load for a single heating season (121 d) for the experimental building was

(8) $$\begin{array}{c} q=3603.8\times 121\times 24\times 3600=37676\;MJ. \end{array}$$

The solar fraction of the system during the first heating season was

(9) $$\begin{array}{c} f_{1}=\frac{11619}{37676}\times 100\%=30.8\%. \end{array}$$

The solar fraction of the system during the second heating season was

(10) $$\begin{array}{c} f_{2}=\frac{22715}{37676}\times 100\%=60.3\%. \end{array}$$

Therefore, the system provided considerably more heat to the building after the dissipating terminals of the solar-powered heating system were changed from radiators to low-temperature floor heaters. This significantly increased the solar fraction and solar utilization efficiency, resulting in substantial energy savings.

## 6. Performance of PV Subsystem

The power-generation subsystem has a PV array covering 6.44 m^2^. During the first heating season, the cumulative solar radiation per unit area of the array was 1901 MJ/m^2^, corresponding to a total solar input of 12242 MJ. Based on (1), the total power generated by the solar array was 1010 MJ, which is equivalent to 280.7 kW·h of power. The intrinsic power consumption of the system was 137.3 kW·h, leaving 143.4 kW·h for household usage. Therefore, the actual PV conversion efficiency of the PV arrays was 8.3%.

During the second heating season, the cumulative solar radiation per unit area of the PV array was 1817 MJ/m^2^, corresponding to a total solar input of 11702 MJ. The total energy generated by the solar array was 942.1 MJ, equivalent to 261.7 kW·h. The intrinsic power consumption of the system was 146 kW·h, leaving 115.7 kW·h for household usage. Therefore, the actual PV conversion efficiency of the PV arrays was 8.1%.

It is clear that the PV conversion efficiency of the system began to decline during the second heating season, mainly reflecting gradually declining output of the PV components. The second reason was that the weather was noticeably poorer during the second heating season, as there were more days of extreme snowy/rainy weather with lower levels of solar radiation. The energy generated by the PV system was greater than the system's intrinsic power consumption. Hence, the system was able to sustain its own power requirements and supply power for household usage, which demonstrated that the system had excellent power-generation performance.

## 7. Biogas-Production Performance

The biogas system uses solar energy and biomass as inputs and produces biogas chemical energy and heat as outputs. This subsystem included evacuated-tube solar collectors, a temperature-controlled chamber, red mud soft-matter biogas bag, heating coil, controller, measurement devices, and data-acquisition device. The evacuated-tube solar collector used in the experiment had an area of 3.85 m^2^ (40 tubes, each 1.8 m long, with an effective solar collector length of 1.66 m and diameter 0.058 m). The angle between the collector's surface and the ground was 45°. The hot water storage tank capacity was 400 L. The temperature-controlled chamber was a 1.9 m × 1.9 m × 2.6 m cuboid. The sides of the chamber were made of coated steel plates sandwiching a 7.5-cm-thick polystyrene board on the outside and 6-cm-thick extruded polystyrene boards on the inside. The bottom of the chamber consisted of a 12-cm-thick extruded polystyrene board, and the top of the chamber was made of coated steel plates sandwiching a 7.5-cm-thick polystyrene board. The chamber was placed on a horizontal surface and was fixed in place using a welded steel frame. The red mud soft-matter biogas bag had an effective storage capacity of 6.4 m^3^ (1.6 m × 1.6 m × 2.5 m). The bag was fitted with feed inlets and gas outlets and was installed inside the temperature-controlled chamber. During operation, feed materials were loaded into the bottom of the bag, while gas accumulated at the top. The feed inlet on the outside of the temperature-controlled chamber was located 1.2 m from the bottom of the biogas bag, and the maximum quantity of feed that could be loaded into the bag was 3.0 m^3^. Aluminum tubes (inner diameter 16 mm, outer diameter 20 mm) were attached to the solar-powered water heater and then secured to the extruded polystyrene boards on the bottom half of the temperature-controlled chamber (inside). The heating coils inside the temperature-controlled chamber had a total length of 40 m, while the aluminum and plastic pipes outside the chamber were insulated with a 3-cm-thick layer of polyethylene insulation.

**Figure 6:**
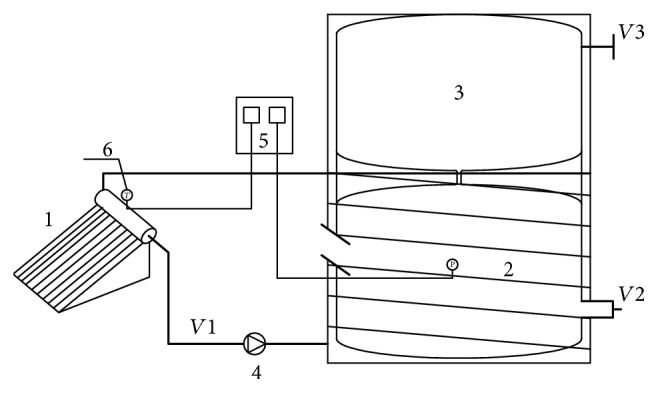
Schematic of solar-heated, thermostatically controlled anaerobic fermentation system for household usage.

In this system, raw materials for fermentation, as specified by the Western China Energy and Environment Research Center (Lanzhou University of Technology, China), were mixed with water at specific ratios. The resulting mixture was then poured into the red mud fermentation bag for soft feed (2 in Figure 6). The evacuated-tube solar collector (1 in Figure 6) absorbs solar radiation and converts this into thermal energy; this is used to heat water, which the water-circulating pump then forces through the coils on the bottom of the fermentation bag and the spiral coils around the chamber (4 in Figure 6). This process transmits the heat from the hot water, via heat radiation and convection, to the fermenting feed slurry, thus maintaining a thermostatic environment that will sustain the anaerobic digestion of biomass. After the fermenting feed slurry has been heated to a specified temperature, the automated temperature control box (5 in Figure 6) turns off the water-circulating pump. If the temperature sensor inside the fermentation bag records that the feed slurry has cooled below the temperature range specified for fermentation, the pump is reactivated to continue heating the feed slurry. In Figure 6, *V*_1_ is a shut-off valve, while *V*_2_ and *V*_3_ are ball valves.

**Figure 7:**
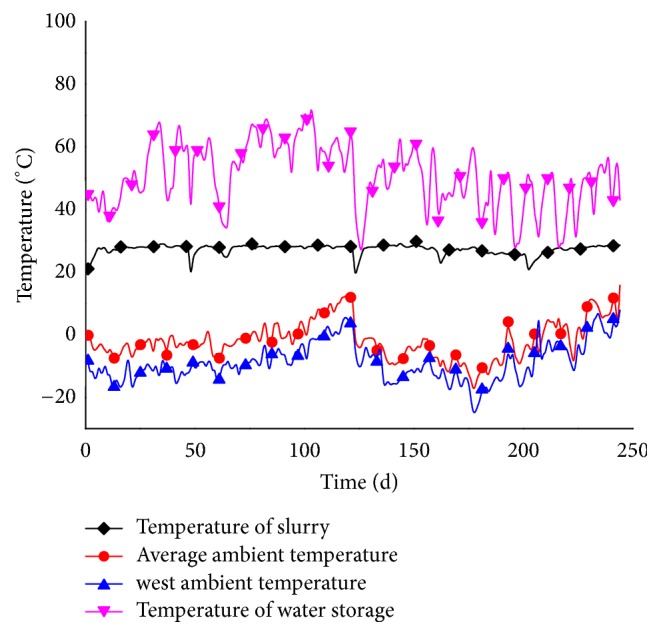
Feed slurry temperature, ambient temperature, and water tank temperature over two heating seasons.

Figure 7 shows the temperature-variation curves of the water tank, feed slurry, and ambient temperature for both winter seasons (242 d). The results show that the biogas digester maintained a temperature within 27 ± 2°C even when the ambient temperature reached its minimum of −25°C. Thus, this system is capable of thermostatic fermentation during the coldest local winter conditions. During the first heating season, cumulative gas production was 114.7 m^3^, with 54.6% average methane content (62.6 m^3^) giving an average daily biogas production of 0.96 m^3^. During the second heating season, cumulative gas production was 120.5 m^3^, with 55.0% average methane content (66.3 m^3^) giving an average daily biogas production of 1.0 m^3^. Therefore, the quantity of biogas produced by this system was sufficient to meet the cooking-gas requirements of a family of four to five members.

## 8. Analysis of Energy Savings

The residents of the experimental home throughout the experimental period primarily consumed energy supplied by the heating-electricity-gas cogeneration system, electrical power from the national power grid, and heat energy generated by the burning of coal. The cogeneration system provided energy from the solar collectors, the PV solar array, and biogas from the solar-powered digester. This combined energy provision was derived solely from solar energy and biomass and was therefore equivalent to the total quantity of fossil fuel energy that was conserved. The energy-saving rate of this system during the first season was calculated using (3), as follows.

(1) The heating provided by the solar collector, *q*_1_, was 11619 MJ. The efficiency of a coal-fired boiler was calculated to be 32% [15], given that the calorific value of standard coal is 29.308 MJ/kg. The energy provided by the solar collector was then equivalent to *m*_1_ = 11619 ÷ (0.32 × 29.308) = 1239 kg of standard coal.

(2) The standard coal consumption of the auxiliary boiler was *m*_2_ = 680 kg.

(3) The power generated by the PV array was *q*_2_ = 280.7 MJ, given that the standard coal coefficient for electrical power is 0.404 kg/kW·h. Therefore, the equivalent amount of coal required to produce this power is *m*_3_ = 113 kg.

(4) Based on local electric power requirements, the average electric power usage of each household was 3.3 kW·h, and the electrical power needed during the heating season was 396 kW·h. Since the heating-electricity-gas cogeneration system was able to supply 143.4 kW·h of power for daily usage, the household still consumed 253 kW·h of power from the national power grid, which is equivalent to consuming 102 kg of standard coal.

(5) The total quantity of biogas produced by the system was 114.7 m^3^, of which 62.6 m^3^ was methane. Given that the calorific value of methane is 35.9 MJ/m^3^ and the heating efficiency of the biogas stove was 75%, the heat released by the burning of biogas was *q*_3_ = 62.6 × 35.9 × 0.75 = 1686 MJ, which is equivalent to *m*_5_ = 1686 ÷ (0.32 × 29.308) = 180 kg of standard coal. The energy saved by the household was then *W*_1_ = *m*_1_ + *m*_3_ + *m*_5_ = 1239 + 113 + 180 = 1532 kg, while the energy consumed by the household was *W*_2_ = *m*_1_ + *m*_2_ + *m*_3_ + *m*_4_ + *m*_5_ = 1239 + 680 + 113 + 102 + 180 = 2314 kg.

The energy-saving rate of the system during the first heating season was therefore

(11) $$\begin{array}{c} \eta =\frac{W_{1}}{W_{2}}\times 100\%=\frac{1532}{2314}\times 100\%=66.2\%. \end{array}$$

Based on the same calculations, the energy saved during the second winter was 2718 kg, while the energy consumed by the household was 3178 kg. This results in an energy-saving rate of 85.5%. Therefore, the energy-saving rate of the system was significantly improved following optimization. This was mainly because the low-temperature floor heaters provided much more heat to the building than did the previous radiator heaters.

## 9. Economic and Environmental Benefits

The system costs comprised the PV power-generation subsystem, including PV arrays; a power inverter; and battery cells (total cost 16000 CNY). The heating subsystem consists of six sets of solar collectors (total cost 11400 CNY). The gas-production subsystem included a thermostatic chamber, a set of solar collectors, and the biogas bag for soft feed (total cost 6900 CNY). Therefore, the initial cost of the system was 34300 CNY. The cogeneration system supplied the low-temperature floor heaters, cooking gas, hot water, winter heating, and electricity and saved an equivalent of 7364 kg in coal a year. If each kg of coal releases 3.67 kg of CO_2_, the reduction in emissions is equivalent to 27.03 t of CO_2_. Based on a coal price of 1500 CNY/t, the savings accumulated over a year would be 11046 CNY, which gives a static payback period of 3.1 yr for this system. Therefore, the heating-electricity-gas cogeneration system studied here is economically viable, energy efficient, and environmentally beneficial.

## 10. Conclusions

The following conclusions were drawn from the operational tests performed on the proposed cogeneration system over two heating seasons under actual working conditions.

(1) After the system was optimized, the average indoor temperature was able to reach 14°C. Average indoor relative humidity was 47% and only fluctuated over a small range.

(2) During the first heating season, the total energy generated by the power-generation subsystem was 280.7 kW·h, of which 137.3 kW·h was consumed by the system's intrinsic power needs, leaving 143.4 kW·h for household usage. Therefore, the actual solar power conversion efficiency of the PV array was 8.3%. During the second heating season, total energy generated by the power-generation subsystem was 261.7 kW·h, with 146 kW·h consumed by the system, leaving 115.7 kW·h for household usage. Therefore, the actual solar power conversion efficiency of the PV array was 8.1%.

(3) Over both heating seasons, the solar-powered thermostatic biogas-generation subsystem was able to maintain a temperature of 27 ± 2°C for thermostatic fermentation inside the biogas digester, even when the ambient temperature reached a minimum of −25°C. Furthermore, the daily biogas production was maintained at approximately 1.0 m^3^ throughout both heating seasons.

(4) After optimizing the system, the energy-saving rate increased from 66.2% to 85.5%. This was primarily because the low-temperature floor heaters provided substantially more heat to the building than the previous radiators. The system was able to save a total of 7,364 kg of standard coal each year, had a static payback period of 3.1 yr, and reduced CO_2_ emissions by 27.03 t each year.

## Figures and Tables

**Table 1:** Types and technical parameters of the measuring instruments.

Measured parameters	Measuring instruments	Technical parameters
Solar radiation on the collector face of solar water heater and PV array	TBQ-2 Pyranometer (Jinzhou Sunshine Technology, Jinzhou, Liaoning)	Range: 0–2,000 Wm^−2^ Sensitivity: 8.963 *μ*V = Wm^−2^ Precision: 2%
Output voltage of PV array	DC voltmeter (Chujing Electric, Wenzhou, Zhejiang)	Range: 0–50 V Precision: 0.5%
Output current of PV array	DC ammeter (Chujing Electric, Wenzhou, Zhejiang)	Range: 0–50 A Precision: 0.5%
Inlet water temperature for space heating Outlet water temperature for space heating Inlet water temperature for digester heating Outlet water temperature for digester heating Ambient temperature	pt100 temperature sensor (Beijing Sailing Technology, Beijing)	Range: −50°C to 100°C Precision: -0.10°C
Flow rate for space heating	LWGY-20 turbine flowmeter (Shanghai Huaman Industrial, Shanghai)	Range: 0.7–7.0 m^3^ = h Precision: -0.45%
Flow rate for digester heating	LWGY-15 turbine flowmeter (Shanghai Huaman Industrial, Shanghai)	Range: 0.4–4.0 m^3^ = h Precision: -0.45%
Quantity of daily consumed coal	Platform balance (Shanghai Shuoheng Electronic Technology, Shanghai)	Minimum scale: 0.2 kg
Daily biogas production	G16 gas meter (Zhejiang Xinlong Instrument, Yongkang, Zhijiang)	Precision: -1.5%
Biogas contents	Gas600 portable biogas analysis (Geotech Instruments, Leamington, UK)	Precision: -2%
Electricity consumed by pumps	Electric energy meter (Wenzhou Libajia Technology, Wenzhou, Zhejiang)	Minimum scale: 0.1 kW·h

**Table 2:** Number of heating days for each mode of heating.

Period	Solar heating (d)	Boiler heating (d)	Solar and boiler heating (d)
2014-2015	55	13	53
2015-2016	90	18	14

**Table 3:** Results of data analysis.

*R* ^2^	Standard error	Partial regression coefficient 1 (*β*_1_)	Partial regression coefficient 2 (*β*_2_)	Intercept
0.633	0.332	2.32	−0.30	5.75

**Table 4:** Calculation of heat consumption by building footprint.

Building footprint	HYC^a^/W (m^2^·K)	CF^b^	Area	*t*_*n*_ − *t*_*w*_ (°C)	HC^c^ (W)
External wall	0.36	0.9	120	16.6	645.4
External window	2	0.9	12	16.6	358.6
Door	3	1.1	5.2	16.6	284.8
Ground	0.47	1	64	16.6	499.3
Roof	1.45	1	64	16.6	1540.5
Total					3328.6

^a^Heat-transfer coefficient. ^b^Correction factor. ^c^Heat consumption.
